# The Effect of Transformational Change on Performance: An Employee’s Stress Appraisals Perspective

**DOI:** 10.3389/fpsyg.2022.897769

**Published:** 2022-05-25

**Authors:** Lei Yan, Li Wang, Xiangdong Shen, Pengfei Li, Jia Guo

**Affiliations:** ^1^School of Business Administration, Jeonbuk National University, Jeonju, South Korea; ^2^School of Business Administration, Anhui University of Finance and Economics, Bengbu, China; ^3^Business School, Changshu Institute of Technology, Changshu, China; ^4^School of Business Administration, Daejeon University, Daejeon, South Korea

**Keywords:** transformational change, in-role performance, cognitive appraisal theory, challenge appraisal, hindrance appraisal

## Abstract

This study aims to determine the specific impact of employees’ perceptions of transformational change on in-role performance and how stress assessment can mediate the relationship between transformational change and in-role performance. According to the cognitive appraisal theory, the same individual has different appraisals of the same stressors, including challenge, and hindrance appraisal. As an important stressor, transformational change also affects individuals differently depending on their assessments. This study integrates employees’ challenge or hindrance appraisal of transformational change into a conceptual model to distinguish between the roles of the two appraisals. It examines it as a mediating mechanism between transformational change and in-role performance. Additionally, 313 employees who recently experienced transformational change were used as samples to test the hypothesis. The results show that transformational change negatively affects employees’ in-role performance; hindrance appraisal negatively mediates the relationship between transformational change and in-role performance, and challenge appraisal positively mediates the relationship between transformational change and in-role performance. The originality and value of this research extend the application of stress appraisals in organizational change management. Research shows that, in the context of major change, employees’ in-role performance is reduced by the impact of transformational change. However, when employees positively appraise organizational change, the negative effects of change are weakened.

## Introduction

The COVID-19 outbreak has generated unprecedented disruptions in global economies, posing a huge threat to human health and property (Chakraborty and Maity, 2020). Additionally, there are significant changes in the way organizations operate, manage, and work (Hartmann and Lussier, 2020). This sudden change is undoubtedly a threat and an opportunity (Kovoor-Misra, 2009). Therefore, most organizations change their reaction to the situation to improve their sustainable competitiveness through continuous changes to enable the organization to develop (Brown and Eisenhardt, 1997; May and Stahl, 2017). It follows that organizational change is necessary for a more stable and prosperous organization during the post-pandemic era (Ågerfalk et al., 2020). In particular, as one of the most representative types of organizational change, transformational change represents a radical change that affects the entire organization, including fundamental changes in organizational culture, practices, and underlying assumptions (Brandt et al., 2019). Such changes affect organizational development and have significant and often underestimated psychological and behavioral implications for individual employees (Lee et al., 2013).

Most of the research covered in previous change reviews focused on how organizations prepare for, implement, and respond to change at the organizational level (Oreg et al., 2011) while ignoring the crucial area of employee responses to change at the individual level (Choi, 2011). Additionally, focusing only on objective measures of change exposure may underestimate the effects of change (Rafferty and Jimmieson, 2017). Because employees’ subjective change experiences may not be consistent with the objective change they face (Loretto et al., 2010). As the main driver of change, how employees react to organizational change is the core of the entire event and the primary determinant of the extent to which any change can succeed (Oreg et al., 2018). Based on this view, recent studies have begun to focus on the significance of individual responses to transformational change in organizational change, especially in subjective perceptions (Rafferty and Jimmieson, 2017). For example, employees’ subjective perception of transformational change leads to their resistance to change and declines their psychological well-being (Rafferty and Jimmieson, 2017). In contrast, employee commitment to organizational change prompts employees to comply with the behavioral requirements for change (Meyer et al., 2007).

Moreover, employees may respond differently to the ongoing changes in their organizations due to individual differences. Some employees feel a growing sense of frustration, alienation, and grief he impact of change and resist it (Smollan, 2012) or are ambivalent and do not change their behaviors (Piderit, 2000). While others regard change as an opportunity to learn and grow and respond enthusiastically to organizational change (Caldwell et al., 2004; Vakola, 2014). These different responses are caused by people’s different perceptions and appraisals of organizational change (Oreg et al., 2011; Çalışkan and Özkoç, 2020). This view agrees with Lazarus and Folkman (1984) that the same environmental stimulus may cause different responses from different individuals depending on how they evaluate and respond to it.

The goal of organizational change is to improve organizational performance. Progress toward this goal is gauged at lower organizational levels regarding employee job performance (Carter et al., 2013). However, little research has been conducted on organizational change’s impact on employees’ job performance. We used the transformational changes of Rafferty and Griffin (2006, p. 1,160) to demonstrate our argument. Transformational change refers to an individual’s perception of the extent to which change involves modifications to an organization’s core systems, including traditional ways of working, values, structures, and strategies (Rafferty and Jimmieson, 2017). According to Lazarus and Folkman (1984) cognitive appraisal theory, an employee’s perception of an event or aspect of the identified environment may be a primary appraisal as a challenge or hindrance. This appraisal is considered one of the psychological mechanisms linking stressors to outcomes (Piccoli et al., 2021), and measures of challenge-hindrance appraisals should be included in occupational risk assessments (Gerich, 2017). Therefore, as a stressor, the impact of transformational change on employees’ job performance may vary according to different cognitive appraisals.

Therefore, this study examines the direct impact of transformational change on in-role performance and the mediating effect of challenge-hindrance appraisals in this process, based on cognitive appraisal theory. Specifically, this study first explores the detrimental effects of transformational change as a stressor on employees’ in-role performance. Second, considering individual differences, individuals may also differ in their appraisal of the same stressor (Lazarus and Folkman, 1984). Thus, the perception of transformational change induces employees’ challenge-hindrance appraisal, and these two stress appraisals may serve as important mediating mechanisms between transformational change and in-role performance. In general, this study differs from previous studies in that we will answer two general organizational management research questions (RQ):

RQ1. How does transformational change affect individual employee in-role performance?

RQ2. What is the intervening role of stress appraisals between transformational change and employee in-role performance?

This study is further comprised of five parts. The second part is the theoretical background after the introduction, which mainly includes the application of theory, hypothesis development, and research framework. The third part introduces the research methods in detail, while the fourth part mainly carries on the empirical analysis and the result explanation of the hypothesis. The last part is the discussion, which mainly introduces the research conclusions, the theoretical and practical implications for researchers and organizations, and gives some substantive suggestions for future research according to the limitations of this study.

## Theoretical Framework and Hypotheses

### Cognitive Appraisal Framework

We used the cognitive appraisal theory of Lazarus and Folkman (1984) to explain the specific impact of transformational change on in-role performance and the mediating mechanism of stress appraisal in this process. Cognitive appraisal theory explains why individuals’ perceptions, appraisals, attitudes, and behaviors differ in the same situation, although an event, condition, or stressor is objectively equal for all (Krohne, 2002). According to this theory, stress is “an individual’s psychological response to a situation, in which there is something at stake for the individual and where the situation taxes or exceeds the individual’s capacity or resources” (LePine et al., 2004). Employees’ perception of transformational change is regarded as a job stressor, which may trigger employees’ psychological stress responses to their job behaviors. In addition, this theory suggests that a person’s perception and primary appraisal of environmental events are crucial in stress management. The primary appraisal is considered to determine whether an event or aspect of the environment is seen as a challenge or hindrance. It is regarded as one of the main psychological mechanisms linking stressors to outcomes (Searle and Auton, 2015). Situations perceived as likely to receive rewards (e.g., recognition and praise) to gain and grow are called challenge appraisals. In contrast, those perceived as threatening one’s potential for wellbeing only by impeding the attainment of goals and development are referred to as hindrance appraisal (Lazarus and Folkman, 1984; Webster et al., 2011).

Research on transformational change has interpreted it as a perceived stressor. However, most empirical studies have focused on the negative impact of transformational change without considering how employees evaluate it from the stress appraisals perspective. It is also important to note that assessing challenges and hindrances need not be mutually exclusive (Horan et al., 2020); thus, individuals can evaluate situations as challenges and hindrances (Searle and Auton, 2015). For example, transformational change is a challenge and threat to individuals because of the potential for mastery and gain in professional development and financial return and the potential for increased role complexity and unclear job demands. Based on this logic, it is important to measure the extent to which a situation is appraised as a challenge or hindrance in assessing one’s appraisals of transformational change. The assumption that everybody makes the same appraisal under the same circumstances (Mazzola and Disselhorst, 2019) and that appraisal can only lead to one of two distinctions (challenge or hindrance) are not consistent with the basic tenets of the appraisal theories of stress (Webster et al., 2011). Therefore, this study takes the next logical step to test the theory directly by measuring each employee’s challenge and hindrance appraisals of change events.

In addition to determining that the individual views transformational change as a challenge and hindrance, cognitive appraisal theory suggests that primary appraisal can influence the types of outcomes a person will experience, such as commitment or performance. Although many organizational studies have documented the role of psychosocial and environmental stressors as determinants of strain and other outcomes, few have examined appraisal (Webster et al., 2011). Primary appraisal is regarded as one of the key intervention mechanisms in the transformational change-outcome relationship. Therefore, this study analyzes primary appraisal as the mediating mechanism of transformational change and employees’ in-role performance.

### Transformational Change, Stress Appraisals, and Performance

Stress-related research has proven negative, positive, curvilinear, and null relationships between different stressors and job performance (Jamal, 2007). However, most studies’ results are in favor of a negative linear relationship (Muse et al., 2003), including individualistic Western contexts (Jamal, 1985) and collectivistic non-Western societies (Singh et al., 2012). With research development, growing research has been devoted to the positive effects of workers placed under demanding stressful conditions (Britt and Jex, 2015). This helps employees perform well in the face of inevitable adverse situations (Ceschi et al., 2017). For example, greater challenging stressors that were beneficial to employees were associated with better performance ratings from their superiors, especially those who felt supported by their organization (Wallace et al., 2009). However, authors reporting such positive stress-performance relationships have been careful to confess that “discussing the positive outcomes that may result from stressful work does not discount the negative effects these stressors may have” (Britt and Jex, 2015, p. 135).

Similarly, as one of the stressors, most research on the impact of organizational change on performance focuses on positive mechanisms to improve the survival of organizations in crisis (Waclawski, 2002; Oreg et al., 2011). As scholars pay attention to individual employees, some believe that change may result in more negative employee outcomes (Bordia et al., 2004). For example, employees’ dissatisfaction after downsizing decreases their overall organizational commitment and individual performance (McKinley et al., 1995). Rafferty and Griffin (2006) defined this kind of change, which damages the original structure of the organization, as a transformational change from the individual subjective perception perspective. Transformational change has resulted in modifications to an organization’s core systems, including traditional ways of working, values, structure, and strategy (Rafferty and Jimmieson, 2017; Çalışkan and Özkoç, 2020). These changes can eliminate employment, bring new roles and responsibilities, and require employees to think and act in new ways and accept new values (Schneider et al., 1996). These new methods undoubtedly reduce the effectiveness of employees’ original resources and threaten their inherent values, thus reducing their opportunities for core work tasks and personal development (Rafferty and Griffin, 2006). Therefore, transformational changes may trigger employees’ negative perceptions, attitudes, and behaviors (Oreg et al., 2011). For example, transformational change leads to increased job insecurity among employees (Çalışkan and Özkoç, 2020) and increased resistance to change (Rafferty and Jimmieson, 2017), which may lead to decreased individual performance.

Thus, as a stressor, the impact of organizational change on performance does not reside in any particular research stream because the different conclusions are attributed to other studies on the link between stressors and performance (Al Hajj, 2020). Recent studies have also proven that the “good” and “bad” effects on performance depend on different types of stressors (LePine et al., 2005). However, some scholars believe that the impact of stressors on results is not the stressors themselves but on how individuals appraise and deal with them (Lazarus and Folkman, 1984). Furthermore, assessing certain environmental events as challenges or hindrances is also an important psychological mechanism in this process (Searle and Auton, 2015). Although most scholars have recognized appraisals as a potential mechanism for performance impact, their research still primarily measured stressors rather than appraisals (Gilboa et al., 2008; Webster et al., 2010; Mazzola and Disselhorst, 2019). With the increasing attention paid to stress research on appraisals, few scholars have verified the impact of appraisals on performance (González-Morales and Neves, 2015; Al Hajj, 2020). Challenge stressors can be appraised as threats or opportunities. Being impacted by appraisals, challenging stressors are not always positively related to performance but only when perceived as opportunities (González-Morales and Neves, 2015). Al Hajj (2020) complemented the research of González-Morales and Neves (2015), demonstrating that hindrance stressors can sometimes be appraised equally as challenges and hindrances, thus having different effects on performance.

Therefore, according to cognitive assessment theory, the primary appraisal is closer to the stress outcome than the stressor itself (Lazarus, 2006). The argument that the primary appraisal of stressors as challenges or hindrances is one of the main psychological mechanisms linking stressors with stress outcomes should also apply to transformational change affecting in-role performance (Rosen et al., 2020; Piccoli et al., 2021). Additionally, the distinction between challenge and hindrance appraisals indicates that different individuals can interpret the same stressors in two ways (Hobfoll, 1989), and the psychological mechanism of the impact of stressors on performance has been recognized by most scholars (Searle and Auton, 2015). However, the relationship between transformational change and performance and the role of the primary appraisal mechanism is unclear. Therefore, we propose that transformational change leads to varying degrees of in-role performance through challenge or hindrance appraisals to fill this research gap. In other words, the challenge and hindrance appraisals of change lead to differences in employees’ in-role performance. An individual with high hindrance appraisal focuses more on the negative aspects of stressors, thereby limiting or interfering with a person’s perceived ability to fulfill job demands or deal with work stressors (Charkhabi, 2019). This reduces the employees’ ability to cope with transformational change, which further reduces employee performance. However, challenge appraisals might be positively related to a person’s perceived ability to fulfill a job demand or deal with situational stressors such as transformational changes (Charkhabi, 2019). This may reduce the negative impact of transformational change on employees’ performance. Based on these arguments, the following hypothesis is proposed:

**H1:** Transformational change will be negatively associated with in-role performance.
**H2:** Challenge appraisal mediates the relationship between transformational change and in-role performance.
**H3:** Hindrance appraisal mediates the relationship between transformational change and in-role performance.

**FIGURE 1:**
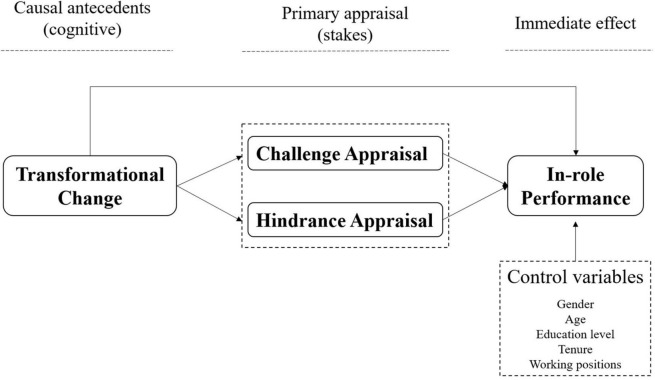
Research model.

The hypotheses were tested using two separate mediation paths based on cognitive appraisal theory. Figure 1 shows the proposed model for the relationship between research variables.

## Research Methods

### Research Approach

In this study, we use the research approach of a questionnaire survey to verify our arguments. The questionnaire survey approach is widely used in empirical research because it is a popular method of connecting a broad sample of the given population at a relatively low cost (Rasool et al., 2021; Zaman et al., 2022). In addition, to efficiently conduct investigations, researchers need to design research questionnaires to quickly collect data from the target population (Hennessy and Patterson, 2011; Rasool et al., 2022). Therefore, we developed the questionnaire according to this approach.

### Questionnaire Development

This study aims to investigate the effect of employees’ perception of transformational change on their in-role performance and the mediating role of employees’ challenge and hindrance appraisals to transformational change in this process. The questionnaire included 15 items from previous studies. The details of the questionnaire items are listed in Appendix Table A1. Furthermore, to ensure that the structure and items of the questionnaire were understandable for the participants, we conducted pilot tests before the questionnaires were distributed. In the pilot test, scholars in related research fields reviewed the questionnaire and gave their suggestions for modification to improve the reliability and validity of the questionnaire.

### Data Collection

To test this study’s hypotheses, we surveyed 380 employees from China between December 10, 2021, and December 20, 2021. Finally, 313 valid responses were obtained with an effective response rate of 82.3%. A demographic analysis of the valid samples found that the proportion of males (37.7%) was much lower than that of females (62.3%), and the majority (62.3%) of the respondents were below 30. The proportion of high school and below, junior college, undergraduate, master’s, and doctoral students accounted for 1.0, 16.0, 44.1, 30.3, and 8.6%, respectively. The majority (67.7%) had fewer than 5 years of tenure. The proportion of managerial positions, marketing, R&D, finance work, administrative work, and other posts accounted for 23.0, 10.5, 31.6, 8.9, 19.8, and 6.1%, respectively. The balanced and diverse work positions of the subjects contribute to the generalization and representativeness of the study. Table 1 summarizes the demographic information of the final sample.

**TABLE 1:** Demographics of survey respondents (*N* = 313).

Demographics	Category	Frequency	%
Gender	Male	118	37.7
	Female	195	62.3
Age	Below 30	195	62.3
	31–40	91	29.0
	41–50	18	5.8
	51 or above	9	2.9
Education	High school and below	3	1.0
	Junior college	50	16.0
	Undergraduate	138	44.1
	Master	95	30.3
	Doctor	27	8.6
Organizational tenure	Below 5 years old	212	67.7
	6–10	56	17.9
	11–15	18	5.8
	16–20	10	3.2
	21 or above	17	5.4
Working positions	Managerial positions	72	23.0
	Marketing	33	10.5
	R&D	99	31.6
	Finance work	28	8.9
	Administrative work	62	19.8
	Other positions	19	6.1

To ensure the validity of the data, first, we determine whether the respondents have experienced an organizational change in the past year or are undergoing organizational change. Second, respondents were informed that the final data would be available only to the research team. Finally, the resulting data were analyzed using IBM SPSS Statistics 25.0 and IBM Amos Graphics 24.0 for statistical testing of measurement models and hypotheses. Amos Graphics specializes in structural equation modeling, visual statistical procedures for path analysis, and confirmatory factor analysis. In the main and intermediary effects, we use structural equation modeling to analyze the impact of transformational change on in-role performance at work and examine the intermediary role of challenge-hindrance appraisals.

### Measures

#### Transformational Change

The transformational change was measured with the 3-item instrument developed by Rafferty and Griffin (2006). A 7-point Likert scale was used for all the change scales. For the transformational change scale, the responses ranged from 1 (not at all) to 7 (a great deal). Employees were asked to respond to the change in items, considering how the work environment has changed over the past year.

#### Stress Appraisals

There is no consensus in the stress appraisal literature on the best way to measure challenge-hindrance appraisals. Some studies have measured employees’ challenge and hindrance appraisals of every stressor separately (Webster et al., 2011; Liu and Li, 2018). Others have used a global approach to measure challenge and hindrance appraisals of job stressors using a multi-item scale (e.g., Searle and Auton, 2015; LePine et al., 2016). In this study, we chose the latter method because job demands exist in multiple demands rather than in isolation (Van Woerkom et al., 2016), which leads to employees’ appraisals of stressors not being isolated from others. Thus, we used Searle and Auton (2015) instrument to measure challenge (e.g., “It/They will help me to learn a lot.”) and hindrance (e.g., “It/They will hinder any achievements I might have). Appraisals with 4-item each. For the validity of the questionnaire, we changed the subject of each item from “It/They” to “In the past year, the transformational change in our organization.” Respondents were asked to rate these items on a 7-point Likert-type scale ranging from 1 (“strongly disagree”) to 7 (“strongly agree”).

#### In-Role Performance

In this study, we used 4-item developed by Williams and Anderson (1991) to measure in-role performance. A sample item is “I adequately complete all of my assigned duties.” Participants scored the items on a 7-point scale ranging from 1 (strongly disagree) to 7 (strongly agree). Self-reports of job performance are widely used in job insecurity studies (e.g., Schreurs et al., 2012).

#### Control Variables

To explore the effectiveness of this study model, several control variables were applied, which may shape in-role performance. Specifically, stemming from the prior research on challenge-hindrance appraisals, this study also investigated commonly used control variables: gender (1 = male, 2 = female), age, education level (ranging from 1: high school and below through 7: Ph.D.), and working positions with the current organization (Ding, 2021). Based on Carter et al. (2013)’ research, organizational tenure also has been included in this study.

## Data Analyses and Results

### Reliability and Validity

Following the two-step approach recommended by Anderson and Gerbing (1988), we first examined the measurement model using principal component factor and confirmatory factor analysis (CFA) to verify the reliability and validity of the instrument and then assessed the structural model.

The validation study of reliability indicated that scale reliability was measured using Cronbach’s alpha (or a) to evaluate internal consistency. According to Nunnally, a scale with a Cronbach’s alpha of ≥ 0.7 is considered reliable (Nunnally, 1994). In this study, the lowest Cronbach’s alpha coefficient for all the constructs was 0.844 (transformational change). In addition, the composite reliability (C.R.) values for each construct ranged from 0.844 to 0.954, all above the suggested threshold of 0.7 (Straub et al., 2004), and exhibited a satisfactory level of reliability. Thus, the scale reliability does not appear to be an issue.

Moreover, a validity test was conducted to assess two types of validity: convergent and discriminant. In this study, the sample’s Kaiser–Meyer–Olkin (KMO) statistic was 0.873, indicating that the data were suitable for factor analysis (Kaiser, 1974). All indicators loaded on the expected factors were higher than 0.6, whereas loadings on other factors for all indicators were lower than 0.4, suggesting good convergent and discriminant validity (Chin et al., 1997). Next, we confirm convergence validity by examining the average variance extracted (AVE) and indicator loadings. As shown in Table 2, all AVE values were higher than the recommended level of 0.5 or above (preferably 0.7 or above) (Fornell and Larcker, 1981). The standard loadings of all items were above the desired threshold of 0.7 and significant at 0.001. This indicates good convergent validity (Chin et al., 1997).

**TABLE 2:** Results of confirmatory factor analysis.

Construct	Indicator	Standard loading	Cronbach’sα	CR	AVE
Transformational change	TC 1	0.832	0.844	0.844	0.644
	TC 2	0.778			
	TC 3	0.796			
Challenge appraisal	CA 1	0.853	0.886	0.888	0.665
	CA 2	0.874			
	CA 3	0.819			
	CA 4	0.706			
Hindrance appraisals	HA 1	0.842	0.897	0.920	0.744
	HA 2	0.905			
	HA 3	0.856			
	HA 4	0.846			
In-role performance	IRP1	0.918	0.954	0.954	0.839
	IRP 2	0.932			
	IRP 3	0.901			
	IRP 4	0.912			

*X^2^/df = 1.376, CFI = 0.991, TLI = 0.989, NFI = 0.968, RMSEA = 0.035.*

Discriminant validity was assessed by examining the correlation coefficients between the constructs under investigation. Hair et al. (1995) suggest that a model meets discriminant validity, provided that the minimum of average variance extracted (AVE’s) is larger than the squares of between-construct correlation coefficients (Hair et al., 1995). The smallest AVE was 0.644, and the maximum square of the between-construct correlation coefficient was 0.204. As shown in Table 3, other between-construct correlation coefficients indicate that AVE’s square roots of AVE (diagonal elements) are greater than the correlation between the structures described in non-diagonal entries, indicating adequate discriminant validity.

**TABLE 3:** Results of analyzing correlations.

Construct	*M*	*SD*	1	2	3	4	5	6	7	8	9
1 Gender	1.623	0.485	–								
2 Age	1.492	0.734	0.101	–							
3 Education	3.297	0.872	0.016	–0.154**	–						
4 Organizational tenure	1.607	1.096	0.028	0.831**	–0.330**	–					
5 Working positions	3.869	2.003	0.157**	–0.041	0.006	–0.038	–				
6 Transformational Change	4.379	1.327	–0.040	0.110	0.039	0.088	–0.010	***0.644***			
7 Challenge appraisal	4.907	1.211	0.004	0.142*	–0.066	0.172**	–0.050	0.258** (0.067)	***0.665***		
8 Hindrance appraisal	3.621	1.173	–0.025	–0.060	–0.013	–0.058	0.042	0.452** (0.204)	–0.076 (0.006)	***0.744***	
9 In-role performance	5.069	1.097	–0.007	0.020	0.022	0.048	–0.087	–0.381** (0.145)	0.187** (0.035)	–0.370** (0.137)	***0.839***

*p < 0.05, **p < 0.01. Diagonal bold italics entries are AVE; the figures in () are the squares of correlation coefficients; all others are correlation coefficients. M, mean; SD, standard deviation.

### Descriptive Statistics and Correlation Analysis

Descriptive statistics of the scales (means and standard deviations) and Pearson’s correlation coefficients for these variables are shown in Table 3. The analysis demonstrated that the control variables (gender, age, education level, organizational tenure, working positions) were not correlated with transformation change and in-role performance (*p* > 0.05), while the age were significantly negatively correlated with education level (*r* = –0.381, *P* < 0.01). Transformational change was negatively correlated with employees’ in-role performance (*r* = –0.381, *p* < 0.01) and positively correlated with challenge-hindrance appraisal (*r* = 0.258, 0.452, *p* < 0.01). Challenge appraisal was positively correlated with employees’ in-role performance (*r* = 0.187, *p* < 0.01). Hindrance appraisal was negatively correlated with employees’ in-role performance (*r* = –0.370, *p* < 0.01). Therefore, it was suitable for further model testing.

### Hypothesis Testing

The proposed hypotheses were tested after examining the measurement validity and reliability. The actual and recommended values of the model fit indices after modifying the original model are listed in Table 4. The fit of the path model was as follows: χ^2^/df = 1.585, CFI = 0.986, GFI = 0.946, TLI = 0.983, NFI = 0.963, and RMSEA = 0.043. The fit indices of the model were better than the recommended thresholds, demonstrating that the model fits the data well and is suitable for further data analysis.

**TABLE 4:** Measures of the model fit.

Fit index	*X*^2^/df	RMSEA	GFI	CFI	NFI	TLI
Recommended range	< 3.84	< 0.08	> 0.90	> 0.90	> 0.90	> 0.90
Model value	1.585	0.043	0.946	0.986	0.963	0.983

*RMSEA, root mean square error of approximation; GFI, goodness of fit index; CFI, comparative fit index; NFI, normed fit index; TLI, non-normed fit index.*

**FIGURE 2:**
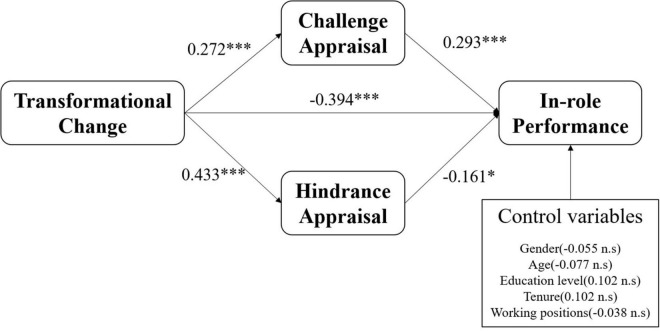
Results of the research model tests. *p < 0.05, ^***^p < 0.001, ns, non-significant at the 0.05 level.

Using a structural equation model, we analyzed the relationship between transformational change and employees’ in-role performance and tested the mediating role of challenge and hindrance appraisals. The standardized path and standardized path coefficients of the structural model are shown in Figure 2. We found that the five paths among the variables and the influence path of hindrance appraisal on in-role performance were significant at *p* < 0.05, and the other paths were significant at *p* < 0.001. These statistical analysis results provide a basis for testing and discussing the research hypotheses.

Table 5 and Figure 2 indicate that all the hypothesized relationships are supported. According to the analysis results, transformational change directly, and negatively impacts employees’ in-role performance (β = –0.394, *P* < 0.001). Therefore, H1 is supported empirically. In addition, H2 and H3 proposed a mediating role of challenge or hindrance appraisals. In structural equation modeling (SEM), the conceptual model of this study belongs to the multiple mediator models, with two mediating variables (challenge appraisal and hindrance appraisal). The analysis of SEM-based bootstrapping can overcome the shortcomings of traditional testing methods, such as the Sobel test, by dealing with small sample sizes and small mediating effect values. It can simultaneously bring multiple mediating variables into the model to gain a deeper understanding of complex management phenomena (Cheung and Lau, 2008). This method can estimate the mediation effect accurately when multiple mediator models are used (MacKinnon et al., 2002). Table 5 shows that the mediating impact of challenge and hindrance appraisal on the relationship between transformational change and employees’ in-role performance is significant, with a 95% bootstrap confidence interval, excluding zero. This finding suggests that challenge and hindrance appraisals mediate the effect of transformational change on employees’ in-role performance. Therefore, H2 and H3 were empirically supported.

**TABLE 5:** Results of hypotheses testing.

Research hypothesis	Path value	S.E.		*p*-value	LLCI	ULCI	Results
H1: TC → IRP	–0.394	0.063		***	–0.524	–0.280	Supported
H2: TC → CA → IRP	0.080	TC→CA	0.063	***	0.038	0.141	Competitive
		CA→IRP	0.052				
H3: TC → HA → IRP	–0.070	TC→HA	0.55	***	–0.128	–0.013	Complementary
		HA→IRP	0.65	*			

*p < 0.05, ***p < 0.001. TC, transformational change; CA, challenge appraisal; HA, hindrance appraisal; IRP, in-role performance; 95% Bootstrap confidence intervals for indirect effect.

## Discussion and Conclusion

### Discussion of Findings

The major purpose of this study is to explore the impact of transformational change on in-role performance and the extent to which the challenge or hindrance appraisals of transformational change mediate the relationship between transformational change and in-role performance. The results indicate that all hypotheses in this study are supported. First, transformational change significantly impacts employees’ in-role performance negatively. This result is consistent with the argument supported by most scholars that stressors have a negative linear relationship with performance (Wallace et al., 2009; Eatough et al., 2011). From the sense-making perspective, transformational changes to the organization’s structure, processes, and culture are commonly associated with disruption to implicit sense-making. This change forces people to shift from implicit sense-making activities to explicit and often taxing information-gathering and processing behaviors (Kuntz and Gomes, 2012). This unavoidable burden leads to an increase in negative attitudes and behaviors among employees.

Second, the mediating effect of stress appraisals on transformational change showed that challenge appraisal played a positive mediating role between transformational change and in-role performance. In contrast, hindrance appraisal played a negative mediating role between transformational change and in-role performance. This finding is explained by the cognitive appraisal theory (Lazarus and Folkman, 1984). According to this theory, the appraisal of transformational change creates positive anticipation of how beneficial the threat will be. Such positive anticipation represents the likelihood that employees will gain or grow during the transformational change process. Therefore, employees with such positive anticipation may be more inclined to face negative stress positively, thus reducing negative emotions, attitudes, and behaviors caused by negative stress events. Conversely, a hindrance appraisal of the threat (e.g., transformational change) forms negative anticipation toward how harmful the threat will be. Such negative expectations undermine or inhibit the coping ability of employees to deal with such threats. Therefore, employees with such negative expectations are expected to report more strain in terms of in-role performance.

### Theoretical Implications

Consistent with the arguments supported by most scholars, this study revealed that stressors (e.g., transformational change) significantly impact employees’ in-role performance negatively. However, compared with previous studies on the effects of organizational change on in-role performance, this study focuses on employees’ subjective evaluations of transformational change rather than objectively measuring existing changes. This personal evaluation explains the theoretical basis of the impact of transformational change on individual performance. Moreover, measuring only objectively existing changes may underestimate the effect of change on performance.

Second, this study successfully confirmed the applicability of the cognitive appraisal framework to explain the impact of transformational change on in-role performance. Few studies have empirically examined the effects of transformational change on employees’ in-role performance, leaving relatively little knowledge in this field. Based on the cognitive appraisal framework, this study explores the impact of transformational change on employees’ in-role performance, expands the research field on the effects of change on employees’ behavior, and provides a new direction for future research.

Third, this study was carried out based on cognitive appraisal theory, and the findings indicate that challenge and hindrance appraisals are indirect predictors of employees’ in-role performance. Challenge and hindrance appraisals mediate the impact of transformational change on employees’ in-role performance. Although previous studies have reported cognitive appraisals as mediating mechanisms (Searle and Auton, 2015; LePine et al., 2016; LePine, 2022), no studies have specifically examined how individual differences in appraisal affect the associations between transformational change and in-role performance. Therefore, these appraisals are regarded as the psychological mechanisms of the impact of change events on employees’ behavior, providing a reference for the individual level of organizational change research in the future. Additionally, this study uses stress appraisals as a mediating factor between change and change outcome, which expands the application field of cognitive appraisal theory and the knowledge system of organizational change.

### Practical Implications

This study shows that transformational change negatively affects employees’ in-role performance. Such negative results may lead to employee resistance to change and a decline in wellbeing (Rafferty and Jimmieson, 2017). Therefore, for the organization’s development, the reduction in employee performance caused by the transformational change may be the most important issue for managers. By understanding the definition of transformational change, the change factors that may lead to employee performance decline are identified, including traditional ways of working, values, structures, and strategies (Rafferty and Griffin, 2006). All these factors may lead to employees’ negative attitudes and behaviors because employees’ negative appraisal of work demands may lead to burnout and reduced engagement (Li et al., 2020). Thus, this study recommends that leaders deepen their understanding of transformational change to actively convey the wellbeing and benefits that change may bring to their employees.

Second, challenge and hindrance appraisals mediated transformational change and employees’ in-role performance. This result indicates that organizational change affects employees in two ways. Employees with high challenge appraisal view the stress of change more positively, thus reducing the harm caused by change. In contrast, employees with high hindrance appraisal may be more biased toward the negative aspects. They may even expand the cognition of the negative effects of transformational change, leading to further enhancement of negative attitudes and behaviors. Therefore, organization managers may distinguish employees who have different appraisal dispositions of change and encourage employees to regard change events as challenges (with potential benefits and growth opportunities) through communication. In addition, during the selection process, it may be more beneficial for the organization to select employees who consider the appraisal of demands related to a particular job as a challenge.

Third, the results of this study show that the difference in individual cognitive appraisal is an important factor leading to different outcomes of transformational change. That is, employees offer different in-role performances through different appraisals of the change. The immediate cause of these appraisals may be dynamic changes in resources, including the loss of resources through disengagement or depletion and gain of resources, which may have different effects on in-role performance (LePine, 2022). Therefore, organizational managers may use these findings to design support and training programs to help employees reduce their detrimental appraisal of transformational change. For example, they can provide employees with sufficient organizational resources to improve their employability, thus lowering their concerns about the future (Çalışkan and Özkoç, 2020). In addition, managers need to actively discuss the negative consequences of employees’ hindrance appraisal to provide corresponding coping strategies and reduce employees’ negative perceptions of the outcomes.

### Limitations and Future Research

This study focuses on the personal cognitive appraisal to explore the impact of change events on employees’ in-role performance and the important psychological mechanism of challenge or hindrance appraisal as the mediating factor. Researchers acknowledge that there are individual differences in the appraisal of stressors; therefore, sample selection may limit the generality of the findings. Therefore, future studies may promote the study of the influence of other stressors on employee behavior by selecting more samples to verify stress appraisals as mediators. Second, to explain the impact of change events on individual behaviors more clearly, future studies may further expand the knowledge of the effect of stressors on employee behavior by combining multiple theories. For example, the conservation of resources theory (Hobfoll, 1989) and equity theory are applied to the relationship between stressors and in-role performance (Al Hajj, 2020). The sense-making perspective (Weick, 1995) and cognitive appraisal theory are devoted to the relationship between organizational change and employee wellbeing (Rafferty and Jimmieson, 2017). In addition, the cognitive appraisal framework consists of a primary appraisal and a secondary appraisal. The primary appraisal represents whether an individual evaluates an event or situation as a challenge or hindrance and pays more attention to the loss and benefit caused by the event. While secondary appraisal involves evaluating one’s capacity to cope or deal with the situation. This concerns evaluations of factors such as the situation and personal resources (Lazarus and Folkman, 1987; LePine, 2022). As an important stress event, transformational change may lead to resource loss or profit of employees. Based on this logic, we focus on how change affects employees’ in-role performance and the role of the primary appraisal mechanism in this process, but do not pay attention to the secondary appraisal of the situation or personal resources as the influencing factor of coping strategies (Obbarius et al., 2021). Future studies should incorporate the secondary appraisal into the empirical research framework. The secondary appraisal perspective of contextual factors and individual differences can help us understand under what conditions employees may appraise stressors as challenges or hindrances so that we can better manage stressors (LePine, 2022). Finally, the impact of organizational change on employee behavior may change over time. Future studies may apply a longitudinal research design to test the short-and long-term implications of transformational change on these outcomes and test the influence of both appraisals in the transformational change and in-role performance link over time.

## Conclusion

This study adds to the field of research on change-induced employee outcomes by investigating the effects of transformational change on employee in-role performance. The results show that an employee’s subjective perception of transformational change significantly negatively impacts employee in-role performance. In addition, as an important competitive mechanism, employee challenge appraisal will alleviate the negative effects of transformational; Conversely, employee hindrance appraisal as a complementary mechanism will amplify the negative impact of transformational change. Therefore, as an important psychological mechanism, stress appraisals play an important role in improving employee in-role performance.

## Data Availability Statement

The raw data supporting the conclusions of this article will be made available by the authors, without undue reservation.

## Ethics Statement

Ethical review and approval was not required for the study on human participants in accordance with the local legislation and institutional requirements. Written informed consent for participation was not required for this study in accordance with the national legislation and the institutional requirements.

## Author Contributions

LY designed the manuscript and drafted the initial manuscript. XS and LW designed the method and drafted the initial manuscript. PL and JG collected the data. All authors discussed the results and contributed to the final manuscript.

## Conflict of Interest

The authors declare that the research was conducted in the absence of any commercial or financial relationships that could be construed as a potential conflict of interest.

## Publisher’s Note

All claims expressed in this article are solely those of the authors and do not necessarily represent those of their affiliated organizations, or those of the publisher, the editors and the reviewers. Any product that may be evaluated in this article, or claim that may be made by its manufacturer, is not guaranteed or endorsed by the publisher.

## Appendix

**TABLE A1:** Research model.

Construct	Item
Transformational change	1. Large scale changes significantly changing your unit’s goals.
	2. Changes that affect my work unit’s structure.
	3. Changes to the values of my work unit.
Challenge appraisal	1. In the past year, the transformational change in our organization will help me to learn a lot.
	2. In the past year, the transformational change in our organization will make the experience educational.
	3. In the past year, the transformational change in our organization will show me I can do something new.
	4. In the past year, the transformational change in our organization will keep me focused on doing well.
Hindrance appraisal	1. In the past year, the transformational change in our organization will hinder any achievements I might have.
	2. In the past year, the transformational change in our organization will restrict my capabilities.
	3. In the past year, the transformational change in our organization will limit how well I can do.
	4. In the past year, the transformational change in our organization will prevent me from mastering difficult aspects of the work.
In-role performance	1. I adequately completes assigned duties.
	2. I fulfills my responsibilities specified in my job description.
	3. I Perform tasks that are expected of him/her.
	4. I meet formal performance requirements of the job.
